# Mechanotransduction in the Brain: Soliton Theory, Piezo Receptors and MR Elastography in Headache Pathophysiology

**DOI:** 10.1002/brb3.71385

**Published:** 2026-05-14

**Authors:** Joan Crespi, Axel Karl Gottfrid Nyman, Erling Tronvik, Lanfranco Pellesi, Igor Petrušić, Tobias Navarro Schröder

**Affiliations:** ^1^ Pain Clinic, Department of Anesthesiology and Intensive Care Medicine St. Olavs University Hospital Trondheim Norway; ^2^ Norwegian Centre for Headache Research (NorHead) NTNU—Norwegian University of Science and Technology Trondheim Norway; ^3^ Department of Neuromedicine and Movement Science NTNU—Norwegian University of Science and Technology Trondheim Norway; ^4^ Department of Neurology and Neurophysiology St. Olavs Hospital, Trondheim University Hospital Trondheim Norway; ^5^ Clinical Pharmacology, Pharmacy, and Environmental Medicine, Department of Public Health University of Southern Denmark Odense Denmark; ^6^ Laboratory For Advanced Analysis of Neuroimages, Faculty of Physical Chemistry University of Belgrade Belgrade Serbia; ^7^ Kavli Institute for Systems Neuroscience NTNU—Norwegian University of Science and Technology Trondheim Norway

**Keywords:** headache, mechanosensitive ion channels, MR elastography, piezo, soliton theory, thermodynamics

## Abstract

**Purpose:**

This narrative review aims to explore how incorporating mechanical and thermal dimensions can complement and extend the classical Hodgkin‐Huxley (HH) model to enrich the understanding of headache and pain pathophysiology.

**Method:**

This review examines the integration of three distinct concepts with the HH model. (1) Soliton Theory: This theory proposes that nerve impulses also propagate as density waves through the lipid bilayer, accounting for mechanical and thermal phenomena in neural signaling that are not addressed by the HH model. (2) Mechanosensitive Piezo Ion Channels: These channels, which are activated by mechanical stimuli like stretch and pressure, are reviewed for their expression in neurons and glial cells and their recognized involvement in pain and primary and secondary headache disorders, including roles in CGRP release and glial activation. (3) Magnetic Resonance Elastography (MRE): This noninvasive imaging modality, which quantifies brain tissue stiffness, is discussed for its translational potential in headache research by detecting subtle biomechanical changes, for example, associated with neuroinflammation.

**Finding:**

Recent insights suggest that mechanical and thermal dimensions contribute significantly to neural function, extending beyond the purely electrochemical focus of the HH model. Soliton theory broadens the HH framework by integrating electromechanical and thermodynamic processes. Piezo channels represent a molecular link between mechanical stimuli and pain signaling pathways relevant to pain. MRE has revealed mechanical alterations in neurological disorders, offering a means to detect biomechanical changes associated with pain and headache‐related disorders.

**Conclusion:**

This integrative perspective reframes headache and pain disorders as involving, among other factors, altered brain mechanics. By bridging molecular (Piezo), biophysical (Soliton theory), and imaging (MRE) domains, this framework opens new avenues for diagnosis, biomarker discovery, and therapeutic innovation in pain and headache research.

**Abbreviations**
AD: Alzheimer's disease
ATP: Adenosine triphosphate
CGRP: Calcitonin gene‐related peptide
CNS: Central nervous system
CSD: Cortical spreading depression
CSF: Cerebrospinal fluid
DRG: Dorsal root ganglia
ED50: Median effective dose
FUS: Focused ULTRASOUND
GsMTx4: Grammostola mechanotoxin 4 (a peptide from tarantula venom)
HH: Hodgkin–huxley
HIF1α: Hypoxia‐inducible factor 1‐alpha
ICP: Intracranial pressure
IIH: Idiopathic intracranial hypertension
IL‐6: Interleukin 6
MRE: Magnetic resonance elastography
MRgFUS: Magnetic resonance‐guided focused ultrasound
MRI: Magnetic resonance Imaging
NF‐κB: Nuclear factor kappa‐light‐chain‐enhancer of activated B cells
NSCs: Neural stem cells
OPCs: Oligodendrocyte progenitor cells
PNS: Peripheral nervous system
PTH: Post‐traumatic headache
SNARE: Soluble N‐ethylmaleimide‐sensitive factor attachment protein receptor
TBI: Traumatic brain injury
TRP: Transient receptor potential

## 1 Introduction

The nervous system has traditionally been understood from an electrochemical perspective, with the Hodgkin‐Huxley model serving as the cornerstone of this perspective (Hodgkin and Huxley 1952). This model, developed in the 1950s, revolutionized neuroscience by describing how action potentials are generated and propagated via voltage‐gated ion channels. However, as our understanding of biological systems has deepened, it has become increasingly clear that this model, while foundational, might be incomplete (Heimburg 2022). For example, it fails to account for the thermodynamic and mechanical properties of biological membranes and tissues, which are recognized as critical to neural function (Appali et al. 2010).

Recent discoveries in mechanobiology, particularly the identification of mechanosensitive ion channels Piezo1 and 2, open new possibilities for understanding how mechanical forces influence neural activity (Martinac 2022). These channels respond to stretch, pressure, and shear stress, converting mechanical stimuli into biochemical signals that regulate a wide range of cellular processes.

Mechanical forces influence neural organization across scales, from a molecular level to a macroscopic level. Magnetic resonance elastography (MRE) has emerged as a powerful imaging modality for assessing the mechanical properties of tissues in vivo (Sack 2022). Originally developed for diagnostics in hepatology (Ehman 2022), MRE is currently used only for research purposes in neurology, where it has revealed disease‐related alterations in brain tissue stiffness such as those associated with tumors, aging, neurodegeneration, and hydrocephalus (Svensson 2023; Bunevicius et al. 2020; Pavuluri et al. 2023; Coelho and Sousa 2022; Fattahi et al. 2016).

This review integrates these diverse strands of research to propose a new framework for understanding the nervous system, incorporating not only electrical and chemical aspects but also mechanical factors rooted in thermodynamic principles. We focus on the implications of this framework in headache disorders, with emphasis on migraine and post‐traumatic headache, highlighting the translational potential of MRE.

### 1.1 The Hodgkin‐Huxley Model: A Foundational But Incomplete Framework

**FIGURE 1:**
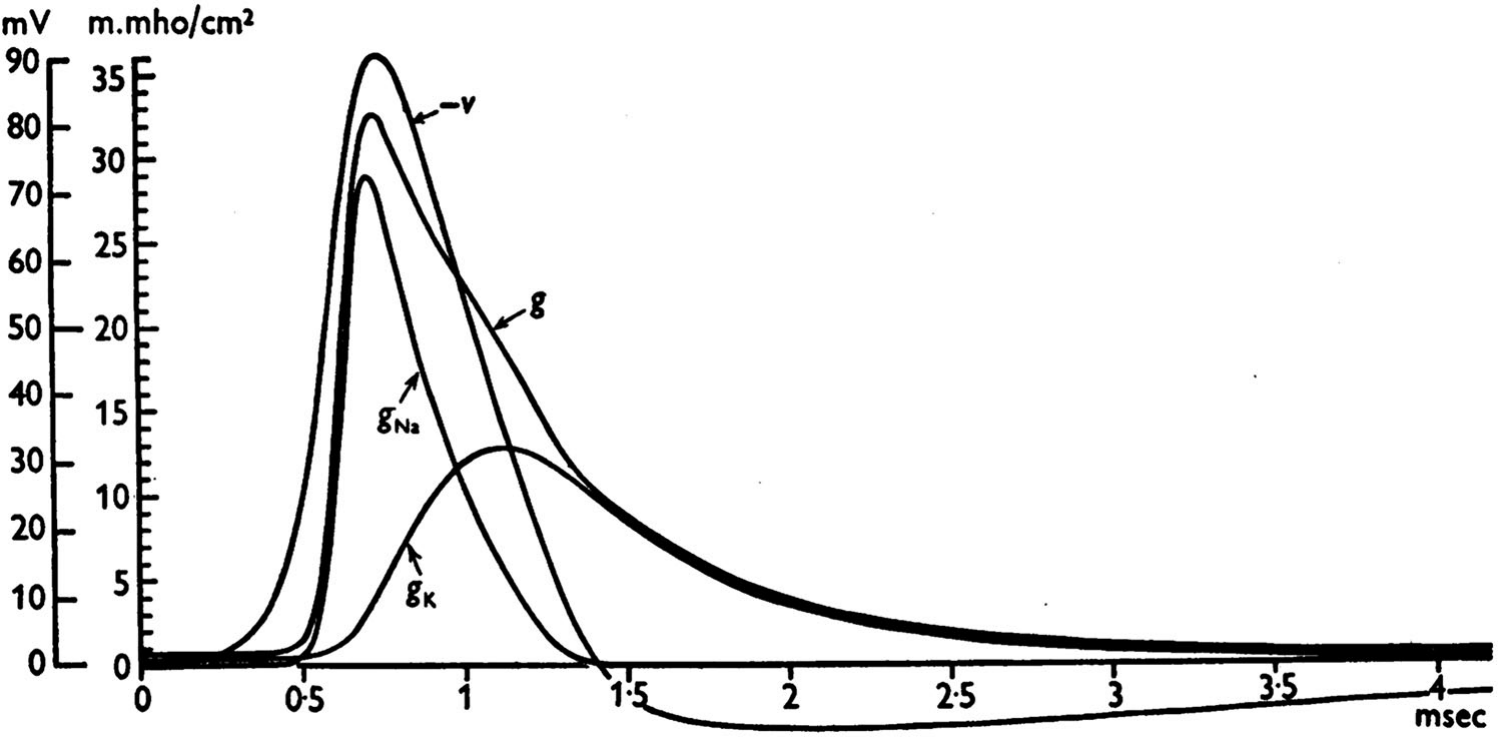
Components of membrane conductance (g) during propagated action potential, adapted from Hodgkin and Huxley (1952) with permission (Hodgkin and Huxley 1952). V: mV, millivolt; m.mho: millimho (mho = 1/Ω); g: conductance; Na: natrium; K: potassium; msec: milliseconds.

The Hodgkin‐Huxley (HH) model, developed by Alan Hodgkin and Andrew Huxley in 1952, is one of the most influential models in the history of neuroscience (Hodgkin and Huxley 1952). It provided the first quantitative description of how action potentials are initiated and propagated in neurons, based on voltage‐gated sodium and potassium channels (Figure 1). This model laid the groundwork for modern electrophysiology and earned its authors the Nobel Prize in Physiology or Medicine in 1963.

The HH model has profoundly shaped how we conceptualize neural function. It introduced the idea that neurons operate as electrical circuits, with ion channels acting as variable resistors and the membrane as a capacitor (Appali et al. 2010). This framework has led to a ligand‐receptor‐centric view of biology, where cellular behavior is understood primarily in terms of chemical signaling and ion flux (Heimburg 2022).

However, while the HH model has been very successful in explaining electrophysiological measurements, it has limitations. From a thermodynamic perspective, the model is incomplete and unable to explain a number of empirical observations. This model treats the membrane as a passive structure and ignores the mechanical and thermal properties of biological tissues. In addition, it does not account for reversible heat changes, mechanical displacements, and changes in membrane thickness and tension that accompany action potentials (Heimburg 2022). In recent years, the importance of mechanical forces in neural signal transduction has increasingly become clear (Griswold et al. 2025; Ucar et al. 2021; Franze 2013; Ryu et al. 2024; Rocha et al. 2022; Abuwarda and Pathak 2020).

These omissions are not trivial. The HH model, while accurate in describing certain electrical phenomena, fails to capture the full complexity of neural signaling. As such, it may lead to incomplete or even misleading interpretations of neurological mechanisms. It is plausible that pathophysiological models (e.g., in migraine) relying solely on ligand‐receptor models may be incomplete if mechanical factors are not considered.

### 1.2 The Soliton Theory: A Thermodynamic Perspective on Neural Signaling

In response to the limitations of the HH model, alternative theories have been proposed that incorporate thermodynamic (and thus mechanical) principles. One of the most compelling is the soliton theory, developed by T. Heimburg and colleagues (Heimburg 2022).

**FIGURE 2:**
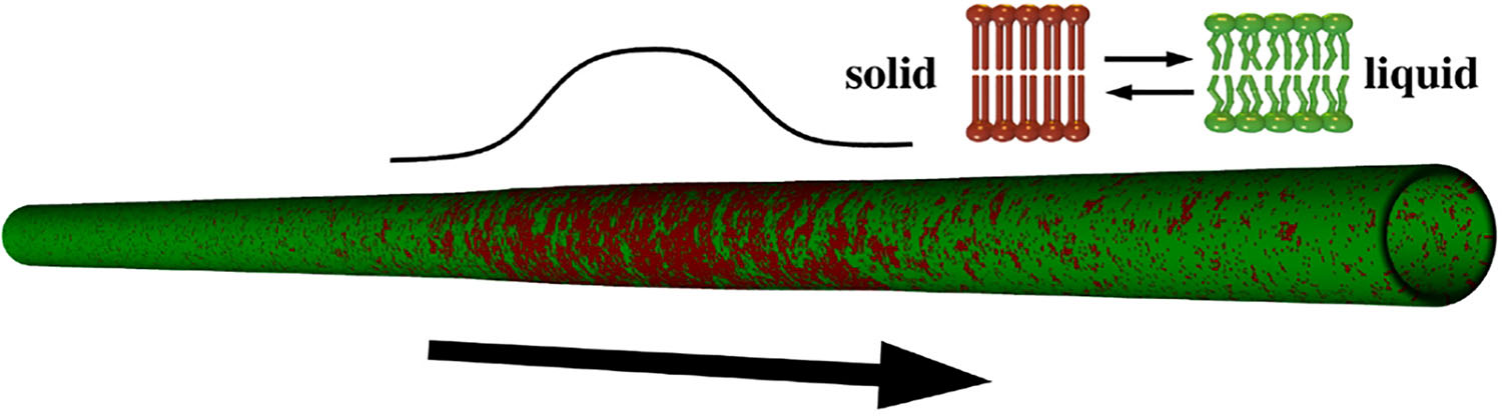
Representation of the electromechanical soliton in a cylinder of a membrane, adapted from Heimburg 2022, with permission (Heimburg 2022). In red: lipids which are more ordered than outside the pulse (green).

According to this theory, nerve impulses are not purely electrical events but are instead adiabatic density pulses (or solitons) that propagate through the lipid bilayer of the neuronal membrane (Appali et al. 2010) (Figure 2). These solitons involve changes in membrane thickness, area, and temperature, and they travel adiabatically (without dissipating energy), much like sound waves in a medium.

The soliton model explains several phenomena that the HH model cannot, including: (1) reversible heat release during action potentials, (2) mechanical displacements of the membrane and (3) the ability of nerves to conduct signals even when ion channels are blocked (Heimburg 2022).

This theory suggests that the nervous system operates not just as an electrical network but as a complex, thermodynamically active system. It emphasizes the role of the lipid bilayer and its phase transitions, proposing that the membrane itself is a dynamic participant in signal propagation. This model can explain not only the effect of anesthetics (e.g., how they can modulate membrane fluidity), alcohol and lithium through their effects on membrane biophysics but also more complex emergent and cooperative phenomena (Heimburg 2022). Such phenomena describe properties of a system that are not present at the level of individual molecules but rather arise from the collective behavior of many components (Heimburg 2022). Emergent phenomena, such as the propagation of waves, depend on macroscopic material constants, not the specific chemistry of individual atoms or molecules. Cooperative phenomena involve a coordinated change in the state of a large number of molecules. Phase transitions like the melting of ice or biological membranes are cooperative, as they cannot be understood by studying a single molecule but instead result from the synchronized interactions of a collective group. Similarly, freezing‐point depression is a cooperative, purely entropic effect that occurs when a solute disrupts the collective network of a solvent's molecules. One example is the Meyer‐Overton correlation, which relates the solubility of an anesthetic in biomembranes (lipophilicity) to its anesthetic potency (Overton and Lipnick 1991). Another example is that increasing hydrostatic pressure can counteract the anesthetic effect in mice (pressure reversal) (Miller et al. 1973). Anesthetics act as melting‐point depressants in nerve membranes, independent of their chemical structure (Heimburg 2007).

Understanding the nervous system through the soliton theory has profound implications. It shifts the focus from ion channels and electrical currents to the physical properties of membranes and tissues. It also provides a framework for integrating mechanical stimuli (such as pressure and stretch) into our understanding of neural function. It is important to emphasize that the soliton theory includes and complements rather than contradicts the HH model.

### 1.3 Piezo Channels

**FIGURE 3:**
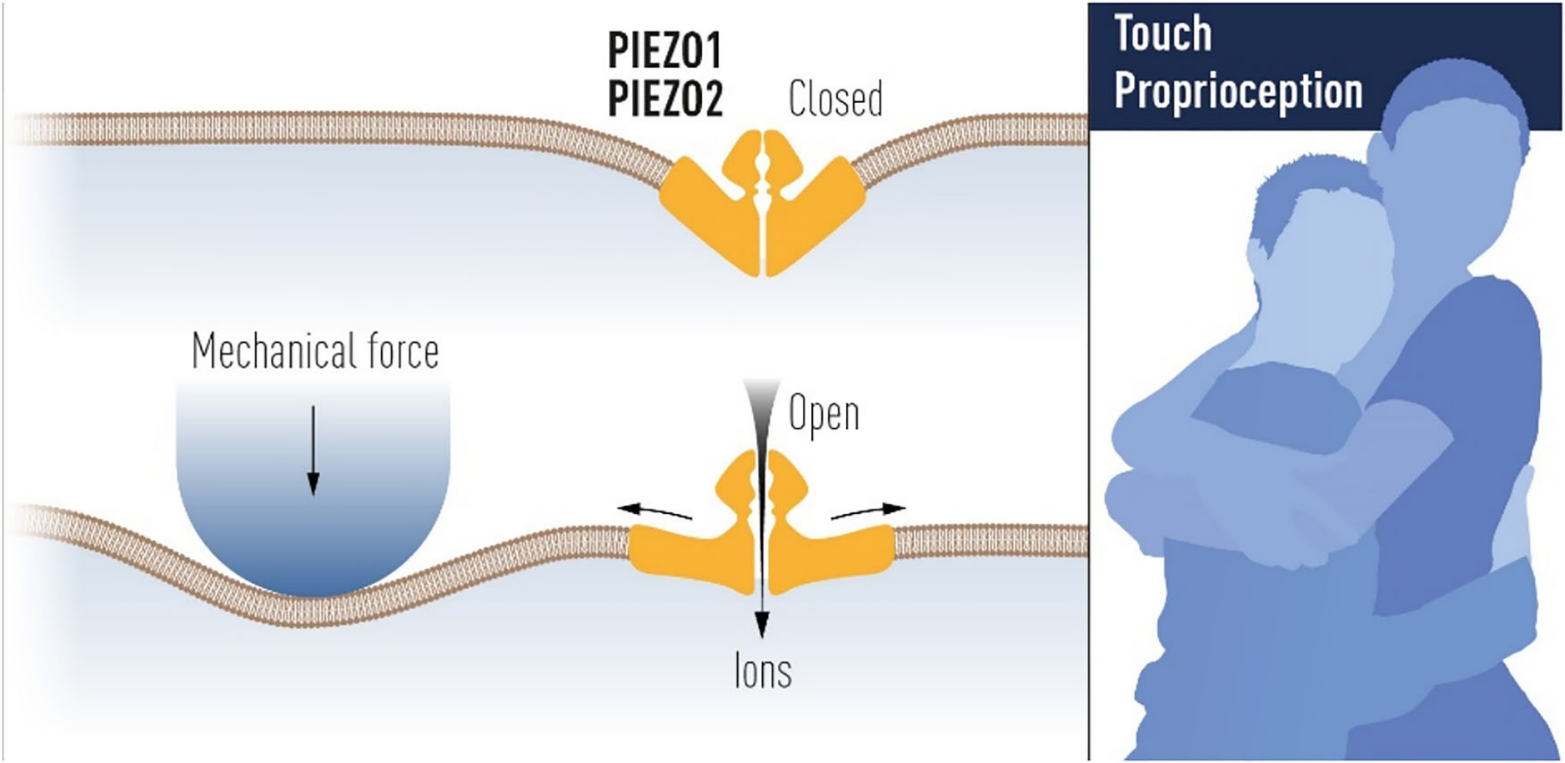
Piezo receptors, with permission from The Nobel Committee for Physiology or Medicine. Ill. Mattias Karlén. (Nobelförsamlingen 2021).

Mechanosensitive ion channels, particularly Piezo1 and 2, have emerged as key players in the transduction of mechanical stimuli into neural signals (Figure 3).

These channels were discovered in 2010 by Ardem Patapoutian and colleagues, a discovery that led to the Nobel Prize in Physiology or Medicine in 2021, shared with David Julius, who discovered temperature‐sensitive Transient Receptor Potential Channels (TRP). The Nobel Committee recognized the significance of this work in revealing how organisms sense touch and temperature.

Piezo channels are large, trimeric proteins that form non‐selective cation channels (Zheng et al. 2023). These channels are embedded in the lipid bilayer and are activated by mechanical forces such as stretch, pressure, and shear stress, leading to the influx of calcium and other ions. This activation triggers a cascade of intracellular events that influence cell behavior (Martinac 2022).

Piezo1 receptors are expressed across a variety of cell types, where they serve as key mechanosensors that translate mechanical stimuli into biochemical signals (Zheng et al. 2023).

Microglia express Piezo1, which regulates neurogenesis and inflammatory responses (Zheng et al. 2023; Liu et al. 2021; Geng et al. 2021). Notably, Piezo1 mediates durotaxis (directed movement or cell growth along a stiffness gradient) toward stiff regions such as amyloid plaques in Alzheimer's disease and can exert either pro‐ or anti‐migratory effects depending on the mechanical context (Hu et al. 2023; Jäntti et al. 2022; Pathak et al. 2014; Zhu et al. 2023). Its role in inflammation is similarly complex: Piezo1 activation may suppress pro‐inflammatory cytokine release via NF‐κB inhibition (Nuclear Factor Kappa‐Light‐Chain‐Enhancer of Activated B Cells) yet, under other conditions, promotes inflammatory signaling (Zhu et al. 2023; Malko et al. 2023).

Astrocytes upregulate Piezo1 upon becoming reactive, for example, in injury or disease (Velasco‐Estevez et al. 2018; Wan et al. 2023). In hippocampal astrocytes, Piezo1 activation promotes ATP (adenosine triphosphate) release and supports adult neurogenesis essential for learning and memory (Chi et al. 2022). Astrocytic Piezo1 can also modulate neural stem cell fate and is an essential regulator in the cell cycle progression of optic nerve head astrocytes (Wan et al. 2023, Li et al. 2022). Piezo1 also regulates astrogliosis and inflammatory responses while also participating in neuron‐astrocyte interactions (Velasco‐Estevez et al. 2018; Wan et al. 2023; Blumenthal et al. 2014).

Oligodendrocytes and their progenitors (OPCs) also express Piezo1, which is activated by age‐related niche stiffening, thereby inhibiting OPC proliferation and differentiation (Segel et al. 2019). Piezo1 knockdown restores myelination potential, highlighting its role as a therapeutic target in demyelinating diseases (Segel et al. 2019). In the peripheral nervous system, Piezo1 regulates Schwann cell myelination in coordination with Piezo2 (Acheta et al. 2022). Mechanosensing is critical for axon growth in the developing brain (Koser et al. 2016), and Piezo channels can inhibit axon regeneration (Song et al. 2019).

Piezo2 channels are broadly expressed in sensory neurons of the dorsal root ganglia (DRG) and trigeminal ganglia (Zheng et al. 2023). They are found in both large low‐threshold mechanoreceptors involved in touch and proprioception and in small nociceptive neurons mediating pain (Zheng et al. 2023). Within neurons, Piezo2 is localized at the plasma membrane, transported along peripheral axons such as the sciatic nerve, and present in central terminals synapsing in the spinal dorsal horn (Zheng et al. 2023). Moreover, Piezo2 is expressed in spinal cord interneurons and motor neurons, suggesting a role in both peripheral and central sensory processing (Zheng et al. 2023). Beyond neurons, Piezo2 is also detected in satellite glial cells, non‐myelinating Schwann cells, and skin structures including Merkel cells and melanocytes, indicating that this mechanosensitive channel participates in neuron‐glia interactions and neurocutaneous signaling relevant for mechanotransduction and pain (Zheng et al. 2023; Moshayedi et al. 2014).

Piezo2, predominantly expressed in dorsal root ganglion (DRG) neurons, has been shown to mediate tactile allodynia (a pain sensation due to a stimulus that does not normally provoke pain), visceral hypersensitivity, and mechanical hyperalgesia in conditions such as osteoarthritis and neuropathic pain (Murthy et al. 2018; Obeidat et al. 2023). Knockout or knockdown of Piezo2 reduces mechanical sensitization in experimental models (Murthy et al. 2018; Obeidat et al. 2023). Piezo1, although more widely expressed in non‐neuronal cells, including keratinocytes, Schwann cells, and satellite glia, also contributes to mechanical hypersensitivity and neuroinflammatory processes, for example, in chemotherapy‐induced neuropathy and osteoarthritis (Wan et al. 2024). Importantly, Piezo channels are also involved in trigeminal nociception, where their activation by pulsatile forces from cranial blood vessels or by inflammatory mediators contributes to migraine pain and/or orofacial hypersensitivity (Mikhailov et al. 2019). Piezo1 and 2 act at the interface of neuronal and glial signaling to promote sensitization, and their contribution is not limited to headache disorders but extends broadly to chronic pain conditions (Wan et al. 2024).

Collectively, Piezo1 and 2 act as widespread mechanosensors in the nervous system, influencing sensory processing and neuroinflammation, and may contribute to the pathophysiology of pain and headache disorders (Zheng et al. 2023).

### 1.4 Piezo Channels in Chronic Pain

In the context of chronic pain, Piezo channels are involved in several processes (Wan et al. 2024). In musculoskeletal pain, Piezo1 activation in chondrocytes leads to calcium influx, mitochondrial dysfunction, and apoptosis, exacerbating joint degeneration in osteoarthritis (Gan et al. 2023). In neuropathic pain, Piezo2 contributes to mechanical allodynia (Terminology 2025). Knockdown of Piezo2 reduces pain sensitivity in models of nerve injury and chemotherapy‐induced neuropathy and after capsaicin application (Murthy et al. 2018; Ferrari et al. 2015).

In visceral pain, Piezo2 mediates hypersensitivity in conditions such as irritable bowel syndrome and interstitial cystitis (Bai et al. 2017; Liu et al. 2023).

In inflammatory pain, Piezo2 is upregulated by cytokines such as IL‐6 and contributes to mechanical hypersensitivity (Liu et al. 2021; Singhmar et al. 2016).

These findings highlight the central role of Piezo channels in chronic pain syndromes and suggest that they might become targets for therapeutic intervention (Wan et al. 2024).

### 1.5 Piezo1 in Post‐Traumatic Brain Injury

Traumatic brain injury (TBI) affects over 3.8 million people in the US every year, and 69 million people are affected worldwide (Maas et al. 2022; Ashina et al. 2019; Langlois et al. 2006). Headache is the most common symptom after mild TBI (Nordhaug et al. 2015). Several cognitive dysfunctions and psychological symptoms are well described in PTH (Ashina et al. 2021). The hippocampus might be involved in the pathophysiology of some of these symptoms (Paterno et al. 2017; Redell et al. 2020, Marzano et al. 2022; McDaid et al. 2021; Atkins 2011; Jorge et al. 2007; Ariza et al. 2006). Neural stem cells (NSCs) in the hippocampus are sensitive to mechanical stress, and their fate can be influenced by the mechanical properties of their environment (Mocciaro et al. 2025).

Piezo1 plays a role in hippocampal NSCs subjected to rapid stretch injury, a model that mimics the mechanical forces experienced during TBI (Mocciaro et al. 2025). This study found that while high‐intensity stretch reduced cell viability, it did not impair proliferation. However, inhibition of Piezo1 using the peptide GsMTx4 (Grammostola Mechanotoxin 4, a peptide from tarantula venom) or Piezo1‐targeting siRNA significantly promoted differentiation toward a neurogenic lineage. This was accompanied by increased expression of microRNAs known to regulate neurogenesis. These findings suggest that Piezo1 acts as a mechanosensitive regulator of NSC fate. Under mechanical stress, Piezo1 activation may inhibit neurogenesis and promote gliosis, contributing to the cognitive deficits and chronic headache often observed after TBI. Therapeutic inhibition of Piezo1 could enhance brain repair by promoting neuronal regeneration and reducing maladaptive glial responses.

### 1.6 Mechanosensitive Receptors in Migraine

A systematic review has highlighted the role of mechanosensitive ion channels in migraine pathophysiology (Della Pietra et al. 2023). One of the hallmark features of migraine is mechanical hypersensitivity, including cutaneous allodynia (Han et al. 2021; Burstein et al. 2000; Harriott and Schwedt 2019). Transient receptor potential (TRP) channels, particularly TRPV1, TRPA1, TRPM3, and TRPV4, are expressed in trigeminal neurons and meningeal afferents and are involved in the release of calcitonin gene‐related peptide (CGRP), a key mediator of migraine pain (Dussor et al. 2014; Julius and Basbaum 2001; Cottarelli et al. 2022, Edelmayer et al. 2012).

It has been reported that the throbbing pain in migraine is not synchronous with arterial pulse (Ahn 2010). Piezo1 is also expressed in trigeminal ganglion neurons and satellite glial cells (Mikhailov et al. 2019; Della Pietra et al. 2023). Activation of Piezo1 by its agonist Yoda1 induces sustained nociceptive firing in meningeal afferents and activates second‐order nociceptive neurons (Mikhailov et al. 2019; Dogorukova et al. 2021). This suggests that Piezo1 activation may contribute to the throbbing pain characteristic of migraine (Mikhailov et al. 2019).

Interestingly, TRPM3 and Piezo1 show sex‐dependent activity, with greater responses observed in female mice (Krivoshein et al. 2022). This may help explain the higher prevalence and severity of migraine in women and underscores the importance of considering sex differences in migraine research and treatment.

Glial cells, once considered merely supportive elements in the central nervous system (CNS), are now recognized as active participants in neural signaling, immune surveillance, and homeostasis (Allen and Lyons 2018). Among glial cells, microglia and astrocytes play essential roles in neuroinflammation, synaptic remodeling, and response to injury (Zheng et al. 2023; Ayata and Schaefer 2020). These cells are not only chemically responsive but also mechanosensitive, capable of detecting and responding to changes in the mechanical properties of their environment (Zheng et al. 2023; Della Pietra et al. 2023). This mechanosensitivity is mediated in part by mechanoreceptors such as Piezo1, which is a critical player in glial function and neuroinflammatory processes (Ayata and Schaefer 2020).

Inflammation has been involved in migraine (Edvinsson et al. 2019). Microglia are the resident immune cells of the CNS, derived from yolk sac progenitors during early embryogenesis (Ayata and Schaefer 2020). They are highly dynamic, constantly surveying brain parenchyma and responding to injury, infection, and pathological changes. One of the most intriguing aspects of microglial biology is their ability to sense mechanical cues in their environment, a property that might have implications in migraine pathophysiology (Della Pietra et al. 2023). Mechanosensing in microglia is mediated by a variety of receptors, including integrins, purinergic receptors, and mechanically gated ion channels such as Piezo1 (Ayata and Schaefer 2020). Piezo is highly expressed in microglia and has been implicated in regulating inflammatory responses through pathways involving HIF1α (Hypoxia‐Inducible Factor 1‐alpha) and endothelin‐1 (Solis et al. 2019).

Astrocytes, the most abundant glial cells in the CNS, also exhibit mechanosensitive properties (Ayata and Schaefer 2020). They respond to changes in tissue stiffness, shear stress, and osmotic pressure, modulating their morphology, gene expression, and secretory profile (Ayata and Schaefer 2020). In models of Alzheimer's disease, Piezo1 expression is upregulated in astrocytes surrounding amyloid plaques, particularly in response to inflammation (Velasco‐Estevez et al. 2018). The activation of Piezo1 in astrocytes leads to calcium influx, which can influence a range of cellular processes, including cytokine production, and modulation of blood‐brain barrier integrity (Ayata and Schaefer 2020). These responses might not only be relevant to neurodegeneration but also to migraine, where astrocyte activation and neuroinflammation are recognized as contributing factors (Ayata and Schaefer 2020; Edvinsson et al. 2019).

One of the most compelling links between glial mechanosensitivity and migraine is the role of Piezo1 in modulating the release of calcitonin gene‐related peptide (CGRP), a neuropeptide that plays a central role in migraine pathophysiology. CGRP is released from trigeminal afferents and glial cells in response to various stimuli, including mechanical stress (Iyengar et al. 2019). Recent studies have shown that activation of Piezo1 by the selective agonist Yoda1 induces CGRP release from meningeal afferents in *ex vivo* preparations (Mikhailov et al. 2019). This finding suggests that mechanical activation of Piezo1 in glial cells could contribute to the initiation or amplification of migraine attacks by promoting CGRP‐mediated neurogenic inflammation (Della Pietra et al. 2023; Dussor 2019).

It has been hypothesized that Piezo1 expression in both microglia and astrocytes may position these cells as potential amplifiers of mechanical signals in the meninges and cortex. This could be relevant during cortical spreading depression (CSD), a phenomenon linked to migraine aura, where mechanical and metabolic changes in the cortex might activate Piezo1 in glial cells. This activation, in turn, could potentially lead to CGRP release and the subsequent activation of trigeminal nociceptors.

The recognition that glial cells are mechanosensitive and contribute to migraine pathophysiology opens new avenues for therapeutic intervention. Targeting Piezo1 and other mechanoreceptors in glial cells could modulate their inflammatory responses, potentially reducing CGRP release and attenuating migraine symptoms.

Pharmacological modulation of Piezo1 is still in its early stages. Compounds like Yoda1 (agonist) and GsMTx4 (inhibitor) provide valuable tools for exploring the functional consequences of Piezo1 (Mocciaro et al. 2025; Della Pietra et al. 2023; Dussor 2019). Inhibiting Piezo1 in glial cells could suppress the release of pro‐inflammatory cytokines and neuropeptides, offering a novel strategy for migraine prevention and treatment. Mechanoreceptors in glial cells, particularly Piezo1, represent a critical interface between mechanical stimuli and neuroinflammatory responses. Their expression in microglia and astrocytes, and their ability to modulate CGRP release, position them as key players in the pathophysiology of migraine. Understanding the mechanisms of glial mechanosensing and its impact on neural circuits offers exciting opportunities for developing new therapeutic strategies.

Furthermore, the lipid composition of glial membranes influences Piezo1 sensitivity. Saturated fatty acids reduce membrane fluidity and inhibit Piezo1 activation, while unsaturated fatty acids restore its function (Romero et al. 2019). This suggests that dietary interventions or lipid‐modifying drugs could indirectly modulate Piezo1 activity and glial mechanosensitivity. According to the soliton theory, such interventions have the capability of changing the thermodynamic properties of lipidic membranes. A study examining the effectiveness of atorvastatin as a prophylactic treatment in chronic migraine, and another study in episodic migraine, are currently ongoing in our center.

Mechanical forces shape neural signaling at multiple levels. During learning, enlargement of dendritic spines can physically push presynaptic boutons, boosting SNARE (soluble *N*‐ethylmaleimide‐sensitive factor attachment protein receptor) assembly and glutamate release without relying on calcium (Ucar et al. 2021). Axons also respond to tension and membrane mechanics: changes in pressure or lipid composition alter their shape and conduction speed (Griswold et al. 2025). A periodic actin–spectrin cytoskeleton acts as a shock absorber, preserving axonal integrity and electrical signaling (Griswold et al. 2025). Outside the cell, perineuronal nets provide structural support and influence plasticity and pain (Li et al. 2024). Together, these findings show that mechanical cues (from cytoskeleton to extracellular matrix) are fundamental to synaptic function, axonal conduction, and processes like memory and pain.

### 1.7 MR Elastography in Brain Mechanics

MRE is a noninvasive imaging technique that estimates the mechanical properties of tissues using motion encoding gradients and tissue‐penetrating, periodic shear waves. The basic principle involves generating low‐frequency mechanical vibrations, capturing the resulting local tissue displacements using MRI, and applying inversion algorithms to estimate the three‐dimensional wave propagation field. This can, in turn, be used to estimate local tissue stiffness (Muthupillai et al. 1995; Mariappan et al. 2010).

**FIGURE 4:**
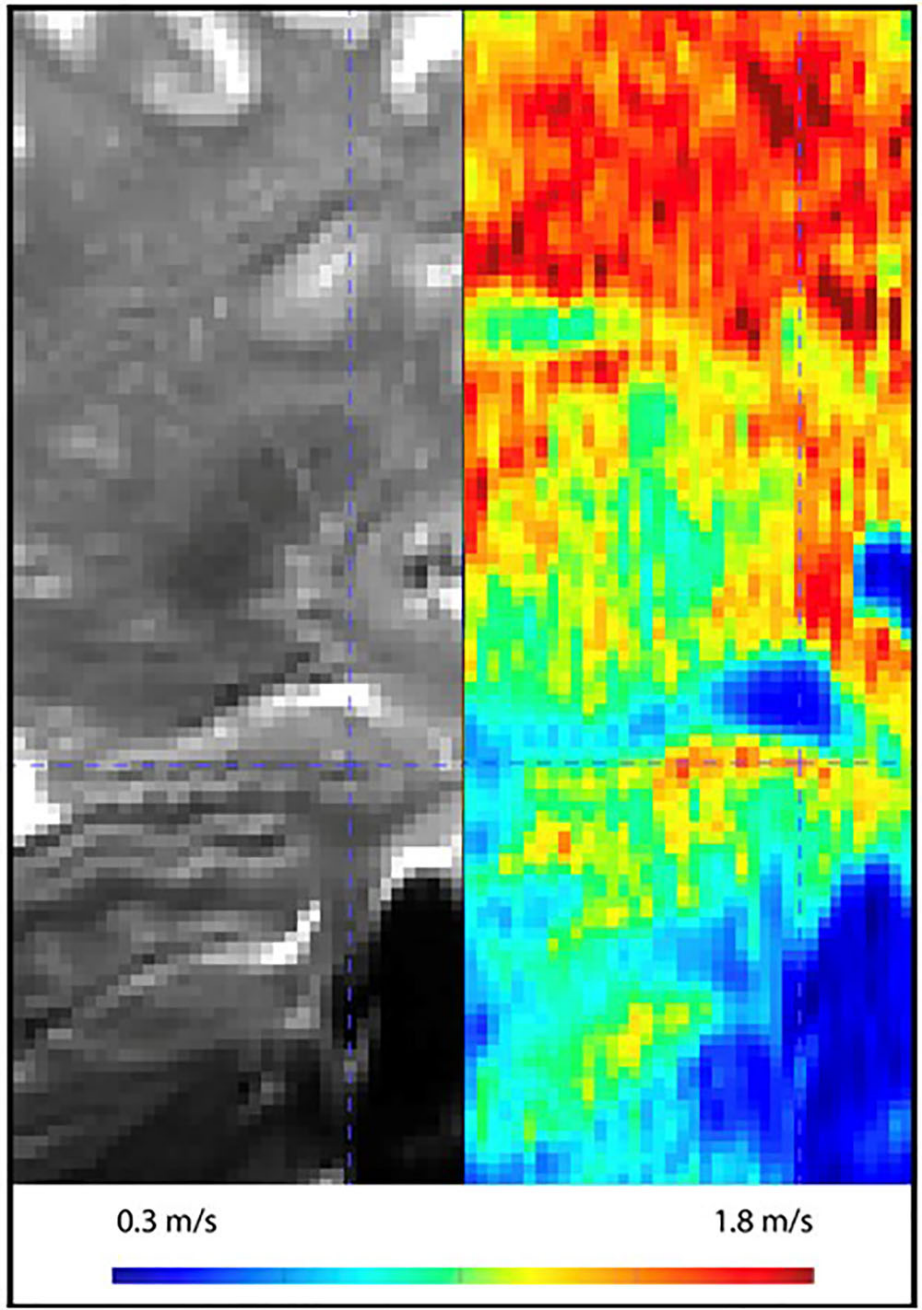
High‐resolution (1.1 mm isotropic) MR elastography magnitude (left side) and shear‐wave speed (right side; units in meters per second) map. Higher shear‐wave speeds are associated with higher tissue stiffness. Crosshair is centered on the head of the hippocampus. Data were acquired on a Siemens Magnetom Terra 7T scanner using a tomoMRE single‐shot spin‐echo echo‐planar imaging (SE‐EPI) sequence with two MRE drivers (Thea Devices), TE 58 ms, and 20–35 Hz vibration frequencies. Wave‐speed maps were estimated using k‐MDEV multi‐frequency inversion according to Meyer et al. 2022. Courtesy of Tobias Navarro Schröder, Trondheim, Norway.

MRE was first developed for liver diagnostics, where it has become a standard tool for assessing liver fibrosis (Ehman 2022). Its success in hepatology has spurred interest in applying MRE to other organs, including the brain (Yin et al. 2018; Murphy et al. 2016) (Figure 4).

MRE might become an important tool to evaluate brain biomechanics in idiopathic intracranial hypertension (IIH). IIH is a condition characterized by elevated intracranial pressure (ICP) and optic disc swelling (Sinclair 2023). IIH shares some pathophysiological features with hydrocephalus, including altered cerebrospinal fluid (CSF) dynamics and increased ICP, both of which can influence brain tissue stiffness (Cogswell 2022). MRE has demonstrated its utility in hydrocephalus by detecting regional brain stiffness changes associated with ventricular enlargement and CSF flow abnormalities (Weickenmeier 2016). These findings suggest that MRE could similarly detect biomechanical alterations in IIH, potentially offering a biomarker for disease severity or treatment response. In IIH, impaired CSF absorption may lead to mechanical stress on brain parenchyma, which MRE can quantify (Cogswell 2022). Furthermore, MRE could help differentiate IIH from other headache disorders by identifying unique stiffness patterns (Cogswell 2022). Given the limitations of current diagnostic tests, including reliance on invasive lumbar puncture, MRE might provide a valuable diagnostic tool for IIH.

Brain MRE has revealed that tissue stiffness decreases with age and in various neurological conditions. For example, in glioblastoma, tumor regions are generally softer than surrounding white matter, and stiffness gradients can extend beyond visible tumor margins (Svensson 2023; Fløgstad Svensson et al. 2022; Pepin et al. 2018). In Alzheimer's disease and other dementias, MRE has shown region‐specific softening, particularly in the medial temporal lobe (Pavuluri et al. 2023; Murphy et al. 2013; Hiscox et al. 2020).

MRE can employ different inversion algorithms that either focus on localized tissue stiffness or account for regional boundary effects. This enables differentiation between intrinsic parenchymal changes and apparent stiffness variations due to atrophy or fluid redistribution. (Sack et al. 2013; McGarry et al. 2015).

The ability of MRE to detect subtle mechanical changes makes it a valuable tool for studying brain function and pathology. It provides a unique window into the biomechanical environment of the brain, complementing traditional imaging modalities (Murphy et al. 2016; Sack et al. 2013). Furthermore, MRE is sensitive to dynamic changes such as capturing real‐time elasticity changes in brain parenchyma in response to the Valsalva maneuver and functional activations akin to fMRI (Herthum et al. 2021).

While MRE offers significant translational potential for detecting biomechanical changes in headache and pain disorders, its current application remains primarily within the research domain. Several technical challenges must be addressed before it can be adopted as a clinical standard. Especially, this includes challenges connected to data analysis required to differentiate between intrinsic tissue stiffness and apparent changes caused by fluid redistribution or atrophy. Additionally, current spatial resolution limits may hinder the detection of very localized mechanical changes in small brain nuclei relevant to pain processing. Unlike its established role in hepatology, brain MRE must also account for the restrictive environment of the skull and the inherent viscoelastic complexity of neural tissue, needing further validation against histological and clinical outcomes before it can serve as a definitive diagnostic tool.

## 2 Discussion

The convergence of soliton theory, Piezo channel biology, and MRE represents a potentially transformative shift in how we understand the nervous system. Traditionally, neuroscience has been dominated by the electrochemical paradigm established by the Hodgkin‐Huxley model. While this model has provided a robust framework for understanding ion channel dynamics and action potential propagation, it has also constrained our thinking to a ligand‐receptor and voltage‐gated perspective. It is becoming increasingly apparent that a more complete picture may also need to consider the complex thermodynamic and mechanical properties of biological tissues (Appali et al. 2010). Mechanical forces are crucial regulators that shape neural signaling and influence neural organization across all scales, from the molecular to the macroscopic level. For instance, a comparative MRI study of 70 primate species reveals that mechanically driven brain folding shapes neocortical organization and behavior more than genetics (Heuer et al. 2025).

Soliton theory challenges this paradigm by proposing that nerve impulses are not merely electrochemical events but are also mechanical and thermodynamic phenomena (Heimburg 2022). This theory aligns with experimental observations of reversible heat release and mechanical displacements during action potentials, phenomena that the HH model cannot explain (Hodgkin and Huxley 1952). It also provides a theoretical foundation for understanding how mechanical forces can influence neural signaling. As stated above, rather than excluding the HH model, soliton theory expands it by integrating the full range of thermodynamic processes.

Magnetic resonance‐guided focused ultrasound (MRgFUS) offers a direct method for manipulating mechanical properties of brain tissue (Meng et al. 2021). With this emerging technology, an array of transducers delivers ultrasonic energy through the intact skull. A combination of CT‐guided correction of acoustic aberration with real‐time MR thermometry allows very precise interventions. By delivering enough energy to heat the tissue to > 56°C, irreversible lesions can be produced. This approach has shown great success in treating essential tremor and Parkinson's disease (Cosgrove et al. 2023; Monteiro et al. 2024). MRgFUS has also been used in patients with therapy‐resistant trigeminal neuralgia to produce a lateral thalamotomy (Gallay et al. 2020). Interestingly, FUS stimulation below the thermoablative threshold can change neuronal excitability and result in perceptual and neurophysiological effects in humans (Lee et al. 2016). Widespread clinical interest in MRgFUS has led to increasing availability of these systems, for instance, the use of an ultrasound‐induced wireless implantable stimulator for pain management (Ultrasound‐Induced Wireless Implantable Stimulator for Adaptive Pain Management 2025). The integration of improved understanding of the mechanical contributions to neural physiology and pathophysiology with an intervention that can precisely and non‐invasively manipulate brain mechanobiology holds great promise for future treatment of headache disorders. Soliton theory provides a more comprehensive rationale for these emerging therapeutic strategies.

Piezo channels offer a molecular mechanism for mechanotransduction in the nervous system. Their discovery and characterization have revealed that cells can directly sense and respond to mechanical stimuli (Martinac 2022). Piezo1 and 2 are now known to play critical roles in touch, proprioception, pain, and neurogenesis. Their expression in neurons, glia, and stem cells highlights their broad relevance to brain function and pathology (Zheng et al. 2023).

MRE provides a translational bridge between these theoretical and molecular insights and clinical practice. By measuring tissue stiffness in vivo, MRE allows us to visualize the mechanical properties of the brain (Murphy et al. 2013). MRE has demonstrated its utility in differentiating between various forms of dementia. Alzheimer's disease, frontotemporal dementia, and dementia with Lewy bodies each exhibit unique patterns of cortical softening (Pavuluri et al. 2023). These findings suggest that MRE can serve as a biomarker for neurodegenerative diseases, complementing traditional imaging and molecular diagnostics.

This MR modality might provide additional insights into the pathophysiology of headache disorders. MRE can detect subtle changes in brain stiffness (for instance, associated with neuroinflammation) that may underlie the pathophysiology of some headache disorders. The use of MRE to assess changes in brain stiffness following a traumatic injury is an interesting area for future research. In combination with ongoing studies on Piezo1, this could provide a foundation for developing novel therapeutic strategies aimed at promoting neurogenesis and reducing gliosis, which may ultimately help in treating PTH. Nowadays there are no clinically available objective prognostic biomarkers in PTH. MRE might be able to discern from an early stage which patients might develop persistent PTH and thus be good candidates for early intervention. This would be advantageous to design pharmacological trials and be able to predict at an early stage which patients should be included.

The integration of these diverse perspectives (electrophysiological, thermodynamic/mechanical, and molecular) offers a more complete understanding of the nervous system. It opens new avenues for research and clinical practice, emphasizing the importance of mechanical forces in brain function and dysfunction.

The proposed framework in this paper establishes a multiscale link between molecular events and macroscopic observations in headache and pain pathophysiology. At the molecular level, Piezo1 and Piezo2 channels function as primary transducers, converting mechanical forces—such as membrane stretch or shear stress—into biochemical signals like calcium influx. These molecular sensors operate within the lipid bilayer, a structure that soliton theory reframes as a dynamic, thermodynamically active medium capable of propagating electromechanical density pulses. Unlike the purely electrical focus of traditional models, soliton theory provides the biophysical rationale for how mechanical and thermal energy moves through neural tissues without dissipation. Finally, MRE might provide the macroscopic readout of this system, quantifying the in vivo stiffness and viscoelastic properties of the brain parenchyma. By detecting subtle mechanical alterations associated with neuroinflammation or structural remodeling, MRE has the potential to bridge the gap between theoretical biophysics and clinical imaging, offering a noninvasive means to observe the cumulative effects of altered mechanosensing and wave propagation in the living brain.

## 3 Conclusion

The classical Hodgkin‐Huxley model has profoundly shaped our understanding of neural signaling, but it is increasingly clear that this model is incomplete. It fails to account for the thermodynamic and mechanical properties of biological tissues, which are now recognized as critical to neural function. Soliton theory offers a compelling alternative, proposing that nerve impulses are adiabatic density pulses that propagate through the lipid bilayer of the membrane. Soliton theory does not replace the HH model; it broadens its perspective by including mechanical dynamics in neural signaling.

Mechanosensitive ion channels, particularly Piezo1 and 2, provide a molecular basis for mechanotransduction in the nervous system. Their discovery has revolutionized our understanding of how cells sense and respond to mechanical stimuli. These channels play key roles in neurogenesis, pain, inflammation, and neurodegeneration.

MRE offers a powerful tool for translating these insights into clinical practice. By measuring tissue stiffness in vivo, MRE provides a unique window into the biomechanical environment of the brain. It has demonstrated utility in diagnosing and differentiating between various neurological conditions. There is a solid biophysical rationale to support that MRE might become an important research tool in headache disorders.

This mechanical framework offers a robust foundation for the development of objective prognostic biomarkers and personalized treatment strategies in headache and pain medicine. MRE may allow for the subtyping of headache disorders by identifying unique stiffness patterns, such as those that might distinguish IIH from chronic migraine. In the context of PTH, MRE could provide an early‐stage biomarker to predict which patients are at risk for persistent symptoms, facilitating more targeted pharmacological trials. Furthermore, identifying Piezo channels as molecular targets opens the door for novel therapeutics, such as specific mechanoreceptor inhibitors or MR‐guided focused ultrasound (MRgFUS), which could noninvasively modulate brain mechanics and neuronal excitability.

The integration of mechanosensing, soliton theory, and MRE provides a holistic view of the brain as a mechanically sensitive and thermodynamically active organ. Mechanosensing via Piezo channels provides the cellular mechanism for sensing physical changes in the environment, while soliton theory offers a biophysical description of how these signals propagate as mechanical waves through membranes. MRE complements these perspectives by providing a translational tool to measure the macroscopic consequences of these processes in patients. Together, they shift the paradigm of headache and pain research from a purely electrochemical model to one that accounts for the physical forces shaping neural function.

## Author Contributions

J.C.V. conceived the idea for the narrative review, conducted the literature search, synthesized the findings, and wrote the manuscript. J.C.V. was responsible for the overall structure, content, and finalization of the article. A.K.G.N. contributed to the development of the concept of the article, scientific writing and revision of the manuscript. E.T. contributed with the scientific elaboration of the concept of this article and provided editorial feedback to improve the final version. L.P. contributed with the scientific elaboration of the concept of this article and provided editorial feedback to improve the final version. I.P. contributed with the scientific elaboration of the concept of this article and provided editorial feedback to improve the final version. T.N.S. contributed by scientifically reviewing the manuscript; contributed with domain‐specific expertise in MRE, assisted in identifying and selecting relevant literature, and contributed to the writing and revision of the MRE‐related sections and the totality of the article.

## Funding

Joan Crespi Vidal has a postdoctoral position at NorHead (Norwegian Centre for Headache Research) funded by NTNU (Norwegian University of Science and Technology) and the Research Council of Norway (NorHead—pr.nr 328615).

## Conflicts of Interest

The authors declare no competing financial or non‐financial interests. This includes the absence of any relevant financial activities or affiliations by immediate family members or close associates that could be perceived to influence the work reported in this manuscript. Tobias Navarro Schröder declares that he has no economic or commercial interests in Magnetic Resonance Elastography (MRE) technology beyond its use in academic research.

## Data Availability

The authors have nothing to report.

## References

[brb371385-bib-0001] Abuwarda, H. , and M. M. Pathak . 2020. “Mechanobiology of Neural Development.” Current Opinion in Cell Biology 66: 104–111. 10.1016/j.ceb.2020.05.012. 32687993 PMC7578076

[brb371385-bib-0002] Acheta, J. , U. Bhatia , J. Haley , et al. 2022. “Piezo Channels Contribute to the Regulation of Myelination in Schwann Cells.” Glia 70, no. 12: 2276–2289. 10.1002/glia.24251. 35903933 PMC10638658

[brb371385-bib-0003] Ahn, A. H. 2010. “On the Temporal Relationship Between Throbbing Migraine Pain and Arterial Pulse.” Headache 50, no. 9: 1507–1510. 10.1111/j.1526-4610.2010.01765.x. 20976872 PMC2965597

[brb371385-bib-0004] Allen, N. J. , and D. A. Lyons . 2018. “Glia as Architects of Central Nervous System Formation and Function.” Science 362, no. 6411: 181–185. 10.1126/science.aat0473. 30309945 PMC6292669

[brb371385-bib-0005] Appali, R. , S. Petersen , and U. van Rienen . 2010. “A Comparison of Hodgkin‐Huxley and Soliton Neural Theories.” Advances in Radio Science 8: 75–79. 10.5194/ars-8-75-2010.

[brb371385-bib-0006] Ariza, M. , J. M. Serra‐Grabulosa , C. Junqué , et al. 2006. “Hippocampal Head Atrophy After Traumatic Brain Injury.” Neuropsychologia 44, no. 10: 1956–1961. 10.1016/j.neuropsychologia.2005.11.007. 16352320

[brb371385-bib-0007] Ashina, H. , H. M. Al‐Khazali , A. Iljazi , et al. 2021. “Psychiatric and Cognitive Comorbidities of Persistent Post‐Traumatic Headache Attributed to Mild Traumatic Brain Injury.” The Journal of Headache and Pain 22, no. 1: 83. 10.1186/s10194-021-01287-7. 34311696 PMC8314480

[brb371385-bib-0008] Ashina, H. , F. Porreca , T. Anderson , et al. 2019. “Post‐Traumatic Headache: Epidemiology and Pathophysiological Insights.” Nature Reviews Neurology 15, no. 10: 607–617. 10.1038/s41582-019-0243-8. 31527806

[brb371385-bib-0009] Atkins, C. M. 2011. “Decoding Hippocampal Signaling Deficits After Traumatic Brain Injury.” Translational Stroke Research 2, no. 4: 546–555. 10.1007/s12975-011-0123-z. 23227133 PMC3514866

[brb371385-bib-0010] Ayata, P. , and A. Schaefer . 2020. “Innate Sensing of Mechanical Properties of Brain Tissue by Microglia.” Current Opinion in Immunology 62: 123–130. 10.1016/j.coi.2020.01.003. 32058296 PMC7067639

[brb371385-bib-0011] Bai, T. , Y. Li , J. Xia , et al. 2017. “Piezo2: A Candidate Biomarker for Visceral Hypersensitivity in Irritable Bowel Syndrome?” Journal of Neurogastroenterology and Motility 23, no. 3: 453–463. 10.5056/jnm16114. 28044050 PMC5503296

[brb371385-bib-0012] Blumenthal, N. R. , O. Hermanson , B. Heimrich , and V. P. Shastri . 2014. “Stochastic Nanoroughness Modulates Neuron‐Astrocyte Interactions and Function via Mechanosensing Cation Channels.” Proceedings of the National Academy of Sciences 111, no. 45: 16124–16129. 10.1073/pnas.1412740111. PMC4234571 25349433

[brb371385-bib-0013] Bunevicius, A. , K. Schregel , R. Sinkus , A. Golby , and S. Patz . 2020. “REVIEW: MR Elastography of Brain Tumors.” NeuroImage: Clinical 25: 102109. 10.1016/j.nicl.2019.102109. 31809993 PMC6909210

[brb371385-bib-0014] Burstein, R. , D. Yarnitsky , I. Goor‐Aryeh , B. J. Ransil , and Z. H. Bajwa . 2000. “An Association Between Migraine and Cutaneous Allodynia.” Annals of Neurology 47: 614–624. 10.1002/1531-8249(200005)47:5<614::AID-ANA9>3.0.CO;2-N. 10805332

[brb371385-bib-0015] Chi, S. , Y. Cui , H. Wang , et al. 2022. “Astrocytic Piezo1‐Mediated Mechanotransduction Determines Adult Neurogenesis and Cognitive Functions.” Neuron 110, no. 18: 2984–2999.e8. 10.1016/j.neuron.2022.07.010. 35963237

[brb371385-bib-0016] Coelho, A. , and N. Sousa . 2022. “Magnetic Resonance Elastography of the Ageing Brain in Normal and Demented Populations: A Systematic Review.” Human Brain Mapping 43, no. 13: 4207–4218. 10.1002/hbm.25891. 35488708 PMC9374877

[brb371385-bib-0017] Cogswell, P. M. 2022. “Features of Idiopathic Intracranial Hypertension on MRI With MR Elastography: Prospective Comparison.” AJNR American Journal of Neuroradiology 43: 250–256. 10.2214/AJR.22.27904 PMC10481645 35822642

[brb371385-bib-0018] Cosgrove, G. R. , N. Lipsman , A. M. Lozano , et al. 2023. “Magnetic Resonance Imaging‐Guided Focused Ultrasound Thalamotomy for Essential Tremor: 5‐Year Follow‐up Results.” Journal of Neurosurgery 138, no. 4: 1028–1033. 10.3171/2022.6.JNS212483. 35932269 PMC10193464

[brb371385-bib-0019] Cottarelli, A. , K. Nikolic , G. D'Andrea , and C. Fusi . 2022. “TRP Channels in Migraine: New Targets for Therapy.” International Journal of Molecular Sciences 23: 12345.36293203

[brb371385-bib-0020] Della Pietra, A. , N. Mikhailov , and R. Giniatullin . 2023. “FM1‐43 Dye Memorizes Piezo1 Activation in the Trigeminal Nociceptive System Implicated in Migraine Pain.” International Journal of Molecular Sciences 24, no. 2: 1688. 36675204 10.3390/ijms24021688 PMC9861983

[brb371385-bib-0021] Dogorukova, E. , A. Della Pietra , and G. Dussor . 2021. “Piezo1 Activation Induces Migraine‐Like Pain via Meningeal Afferents.” Journal of Headache and Pain 22: 45.34030630

[brb371385-bib-0022] Dussor, G. 2019. “New Discoveries in Migraine Mechanisms and Therapeutic Targets.” Current Opinion in Physiology 11: 116–124. 10.1016/j.cophys.2019.10.013. 31815209 PMC6897325

[brb371385-bib-0023] Dussor, G. , J. Yan , J. Y. Xie , M. H. Ossipov , D. W. Dodick , and F. Porreca . 2014. “Targeting TRP Channels for Novel Migraine Therapeutics.” ACS Chemical Neuroscience 5: 1085–1096. 10.1021/cn500083e. 25138211 PMC4240253

[brb371385-bib-0024] Edelmayer, R. M. , L. N. Le , J. Yan , et al. 2012. “Activation of TRPA1 on Dural Afferents: A Potential Mechanism of Headache Pain.” Pain 153: 1949–1958. 10.1016/j.pain.2012.06.012. 22809691 PMC3413768

[brb371385-bib-0025] Edvinsson, L. , K. A. Haanes , and K. Warfvinge . 2019. “Does Inflammation Have a Role in Migraine?” Nature Reviews Neurology 15: 483–490. 10.1038/s41582-019-0216-y. 31263254

[brb371385-bib-0026] Ehman, R. L. 2022. “Magnetic Resonance Elastography: From Invention to Standard of Care.” Abdominal Radiology 47: 3028–3036. 10.1007/s00261-022-03597-z. 35852570 PMC9538645

[brb371385-bib-0027] Fattahi, N. , A. Arani , A. Perry , et al. 2016. “MR Elastography Demonstrates Increased Brain Stiffness in Normal Pressure Hydrocephalus.” AJNR American Journal of Neuroradiology 37, no. 3: 462–467. 10.3174/ajnr.A4560. 26542235 PMC4792773

[brb371385-bib-0028] Ferrari, L. F. , O. Bogen , P. G. Green , and J. D. Levine . 2015. “Contribution of Piezo2 to Endothelium‐Dependent Pain.” Molecular Pain 11: 65. 10.1186/s12990-015-0068-4. 26497944 PMC4619430

[brb371385-bib-0029] Fløgstad Svensson, S. , E. Fuster‐Garcia , A. Latysheva , et al. 2022. “Decreased Tissue Stiffness in Glioblastoma by MR Elastography Is Associated With Increased Cerebral Blood Flow.” European Journal of Radiology 147: 110136. 10.1016/j.ejrad.2021.110136. 35007982

[brb371385-bib-0030] Franze, K. 2013. “The Mechanical Control of Nervous System Development.” Development (Cambridge, England) 140, no. 15: 3069–3077. 10.1242/dev.079145. 23861056

[brb371385-bib-0031] Gallay, M. N. , D. Moser , and D. Jeanmonod . 2020. “MR‐Guided Focused Ultrasound Central Lateral Thalamotomy for Trigeminal Neuralgia.” Frontiers in Neurology 11: 271. 10.3389/fneur.2020.00271. 32425870 PMC7212452

[brb371385-bib-0032] Gan, D. , C. Tao , X. Jin , et al. 2023. “Piezo1 Activation Accelerates Osteoarthritis Progression and the Targeted Therapy Effect of artemisinin.” Journal of Advanced Research 62: 105–117. 10.1016/j.jare.2023.09.040. 37758057 PMC11331168

[brb371385-bib-0033] Geng, J. , Y. Shi , J. Zhang , et al. 2021. “TLR4 Signalling via Piezo1 Engages and Enhances the Macrophage Mediated Host Response During Bacterial Infection.” Nature Communications 12: 3519. 10.1038/s41467-021-23683-y. PMC8192512 34112781

[brb371385-bib-0034] Griswold, J. M. , M. Bonilla‐Quintana , R. Pepper , et al. 2025. “Membrane Mechanics Dictate Axonal Pearls‐on‐a‐String Morphology and Function.” Nature Neuroscience 28, no. 1: 49–61. 10.1038/s41593-024-01813-1. 39623218 PMC11706780

[brb371385-bib-0035] Han, S. M. , K. M. Kim , S.‐J. Cho , et al. 2021. “Prevalence and Characteristics of Cutaneous Allodynia in Probable Migraine.” Scientific Reports 11: 2467. 33510340 10.1038/s41598-021-82080-z PMC7844001

[brb371385-bib-0036] Harriott, A. M. , and T. J. Schwedt . 2019. “Mechanisms of Migraine Chronification.” Current Opinion in Neurology 32: 292–298.30720478

[brb371385-bib-0037] Heimburg, T. 2007. Thermal Biophysics of Membranes. Berlin, Germany: Wiley VCH. 10.1002/9783527611591.

[brb371385-bib-0038] Heimburg, T. 2022. “The Thermodynamic Soliton Theory of the Nervous Impulse and Possible Medical Implications.” Progress in Biophysics and Molecular Biology 173: 24–35. 10.1016/j.pbiomolbio.2022.05.007. 35640761

[brb371385-bib-0039] Herthum, H. , M. Shahryari , H. Tzschätzsch , et al. 2021. “Real‐Time Multifrequency MR Elastography of the Human Brain Reveals Rapid Changes in Viscoelasticity in Response to the Valsalva Maneuver.” Frontiers in Bioengineering and Biotechnology 9: 666456. 10.3389/fbioe.2021.666456. 34026743 PMC8131519

[brb371385-bib-0040] Heuer, K. , N. Traut , L. Aristide , et al. “Principles of Neocortical Organisation and Behaviour in Primates.” bioRxiv, July 22, 2025. 10.1101/2025.07.17.665410.

[brb371385-bib-0041] Hiscox, L. V. , C. L. Johnson , M. D. J. Mcgarry , et al. 2020. “Mechanical Property Alterations Across the Cerebral Cortex Due to Alzheimer's Disease.” Brain Communications 2, no. 1:fcz049. 10.1093/braincomms/fcz049. 31998866 PMC6976617

[brb371385-bib-0042] Hodgkin, A. L. , and A. F. Huxley . 1952. “A Quantitative Description of Membrane Current and Its Application to Conduction and Excitation in Nerve.” The Journal of Physiology 117: 500–544. 10.1113/jphysiol.1952.sp004764. 12991237 PMC1392413

[brb371385-bib-0043] Hu, J. , Q. Chen , H. Zhu , et al. 2023. “Microglial Piezo1 Senses A? Fibril Stiffness to Restrict Alzheimer's Disease.” Neuron 111: 15–29.e8. 10.1016/j.neuron.2022.10.021. 36368316

[brb371385-bib-0044] Iyengar, S. , K. W. Johnson , M. H. Ossipov , and S. K. Aurora . 2019. “CGRP and the Trigeminal System in Migraine.” Headache 59, no. 5: 659–681. 10.1111/head.13529. 30982963 PMC6593989

[brb371385-bib-0045] Jäntti, H. , V. Sitnikova , Y. Ishchenko , et al. 2022. “Microglial Amyloid Beta Clearance Is Driven by PIEZO1 Channels.” Journal of Neuroinflammation 19, no. 1: 147. 10.1186/s12974-022-02486-y. 35706029 PMC9199162

[brb371385-bib-0046] Jorge, R. E. , L. Acion , S. E. Starkstein , and V. Magnotta . 2007. “Hippocampal Volume and Mood Disorders After Traumatic Brain Injury.” Biological Psychiatry 62, no. 4: 332–338. 10.1016/j.biopsych.2006.07.024. 17123480

[brb371385-bib-0047] Julius, D. , and A. I. Basbaum . 2001. “Molecular Mechanisms of Nociception.” Nature 413: 203–210. 10.1038/35093019. 11557989

[brb371385-bib-0048] Koser, D. E. , A. J. Thompson , S. K. Foster , et al. 2016. “Mechanosensing Is Critical for Axon Growth in the Developing Brain.” Nature Neuroscience 19, no. 12: 1592–1598. 10.1038/nn.4394. 27643431 PMC5531257

[brb371385-bib-0049] Krivoshein, A. V. , A. Della Pietra , and G. Dussor . 2022. “Sex Differences in TRPM3 and Piezo1 Channel Function in Migraine Models.” Frontiers in Pain Research 3: 100.

[brb371385-bib-0050] Langlois, J. A. , W. Rutland‐Brown , and M. M. Wald . 2006. “The Epidemiology and Impact of Traumatic Brain Injury: A Brief Overview.” The Journal of Head Trauma Rehabilitation 21, no. 5: 375–378. 10.1097/00001199-200609000-00001. 16983222

[brb371385-bib-0051] Lee, W. , H.‐C. Kim , Y. Jung , et al. 2016. “Transcranial Focused Ultrasound Stimulation of Human Primary Visual Cortex.” Scientific Reports 6, no. 1:34026. 10.1038/srep34026. 27658372 PMC5034307

[brb371385-bib-0052] Li, J. , Y. Zhang , Z. Lou , et al. 2022. “Magnetic Nanobubble Mechanical Stress Induces the Piezo1‐Ca(2+)‐BMP2/Smad Pathway to Modulate Neural Stem Cell Fate and MRI/Ultrasound Dual Imaging Surveillance for Ischemic Stroke.” Small 18, no. 23: e2201123. 10.1002/smll.202201123. 35555970

[brb371385-bib-0053] Li, X. , X. Wu , T. Lu , et al. 2024. “Perineuronal Nets in the CNS: Architects of Memory and Potential Therapeutic Target in Neuropsychiatric Disorders.” International Journal of Molecular Sciences 25, no. 6: 3412. 10.3390/ijms25063412. 38542386 PMC10970535

[brb371385-bib-0054] Liu, H. , W. Bian , D. Yang , M. Yang , and H. Luo . 2021. “Inhibiting the Piezo1 Channel Protects Microglia From Acute Hyperglycaemia Damage Through the JNK1 and mTOR Signalling Pathways.” Life Sciences 264: 118667. 10.1016/j.lfs.2020.118667. 33127514

[brb371385-bib-0055] Liu, L. , Y. Zhao , W. An , et al. 2023. “Piezo2 Channel Upregulation Is Involved in Mechanical Allodynia in CYP‐Induced Cystitis Rats.” Molecular Neurobiology 60, no. 9: 5000–5012. 10.1007/s12035-023-03386-9. 37227654 PMC10415424

[brb371385-bib-0056] Maas, A. I. R. , D. K. Menon , G. T. Manley , et al. 2022. “Traumatic Brain Injury: Progress and Challenges in Prevention, Clinical Care, and Research.” Lancet Neurology 21, no. 11: 1004–1060. 10.1016/S1474-4422(22)00309-X. 36183712 PMC10427240

[brb371385-bib-0057] Malko, P. , X. Jia , I. Wood , and L. H. Jiang . 2023. “Piezo1 Channel‐Mediated Ca(2+) Signaling Inhibits Lipopolysaccharide‐Induced Activation of the NF‐?B Inflammatory Signaling Pathway and Generation of TNF‐? And IL6 in Microglial Cells.” Glia 71, no. 4: 848–865. 10.1002/glia.24311. 36447422

[brb371385-bib-0058] Mariappan, Y. K. , K. J. Glaser , and R. L. Ehman . 2010. “Magnetic Resonance Elastography: A Review.” Clinical Anatomy 23, no. 5: 497–511. 10.1002/ca.21006. 20544947 PMC3066083

[brb371385-bib-0059] Martinac, B. 2022. “2021 Nobel Prize for Mechanosensory Transduction.” Biophysical Reviews 14, no. 1: 15–20. 10.1007/s12551-022-00935-9. 35340591 PMC8921412

[brb371385-bib-0060] Marzano, L. A. S. , F. L. M. De Castro , C. A. Machado , et al. 2022. “Potential Role of Adult Hippocampal Neurogenesis in Traumatic Brain Injury.” Current Medicinal Chemistry 29, no. 19: 3392–3419. 10.2174/0929867328666210923143713. 34561977

[brb371385-bib-0061] McDaid, J. , C. A. Briggs , N. M. Barrington , D. A. Peterson , D. A. Kozlowski , and G. E. Stutzmann . 2021. “Sustained Hippocampal Synaptic Pathophysiology Following Single and Repeated Closed‐Head Concussive Impacts.” Frontiers in Cellular Neuroscience 15: 652721. 10.3389/fncel.2021.652721. 33867941 PMC8044326

[brb371385-bib-0062] Mcgarry, M. D. J. , C. L. Johnson , B. P. Sutton , et al. 2015. “Suitability of Poroelastic and Viscoelastic Mechanical Models for High and Low Frequency MR Elastography.” Medical Physics 42, no. 2: 947–957. 10.1118/1.4905048. 25652507 PMC4312344

[brb371385-bib-0063] Meng, Y. , K. Hynynen , and N. Lipsman . 2021. “Applications of Focused Ultrasound in the Brain: From Thermoablation to Drug Delivery.” Nature Reviews Neurology 17, no. 1: 7–22. 10.1038/s41582-020-00418-z. 33106619

[brb371385-bib-0063a] Meyer, T. , S. Marticorena Garcia , H. Tzschätzsch , et al. 2022. “Comparison of inversion methods in MR elastography: An open‐access pipeline for processing multifrequency shear‐wave data and demonstration in a phantom, human kidneys, and brain.” Magnetic Resonance in Medicine 88, no. 4: 1840–1850. 10.1002/mrm.29320. 35691940

[brb371385-bib-0064] Mikhailov, N. , J. Leskinen , I. Fagerlund , et al. 2019. “Mechanosensitive Meningeal Nociception via Piezo Channels: Implications for Pulsatile Pain in Migraine?” Neuropharmacology 149: 113–123. 10.1016/j.neuropharm.2019.02.015. 30768945

[brb371385-bib-0065] Miller, K. W. , W. D. Paton , R. A. Smith , and E. B. Smith . 1973. “The Pressure Reversal of General Anesthesia and the Critical Volume Hypothesis.” Molecular Pharmacology 9, no. 2: 131–143. 10.1016/S0026-895X(25)13842-X. 4711696

[brb371385-bib-0066] Mocciaro, E. , M. Kidd , K. Johnson , et al. 2025. “Mechanosensitive Ion Channel Piezo1 Modulates the Response of Rat Hippocampus Neural Stem Cells to Rapid Stretch Injury.” PLoS ONE 20, no. 5:e0323191. 10.1371/journal.pone.0323191. 40359437 PMC12074584

[brb371385-bib-0067] Monteiro, J. D. S. , B. B. E Silva , R. R. De Oliveira , et al. 2024. “Magnetic Resonance‐Guided Focused Ultrasound Ventral Intermediate Thalamotomy for Tremor‐Dominant Parkinson's Disease: A Systematic Review and Meta‐Analysis.” Neurosurgical Review 47, no. 1: 701. 10.1007/s10143-024-02948-2. 39331247

[brb371385-bib-0068] Moshayedi, P. , G. Ng , J. C. F. Kwok , et al. 2014. “The Relationship Between Glial Cell Mechanosensitivity and Foreign Body Reactions in the Central Nervous System.” Biomaterials 35, no. 13: 3919–3925. 10.1016/j.biomaterials.2014.01.038. 24529901

[brb371385-bib-0069] Murphy, M. C. , J. Huston , C. R. Jack , et al. 2013. “Measuring the Characteristic Topography of Brain Stiffness With Magnetic Resonance Elastography.” PLoS ONE 8, no. 12:e81668. 10.1371/journal.pone.0081668. 24312570 PMC3847077

[brb371385-bib-0070] Murphy, M. C. , J. Huston , C. R. Jack , et al. 2016. “Measuring the Characteristic Topography of Brain Stiffness With Magnetic Resonance Elastography.” PLoS ONE 8, no. 12:e81668. 10.1371/journal.pone.0081668. PMC3847077 24312570

[brb371385-bib-0071] Murthy, S. E. , M. C. Loud , I. Daou , et al. 2018. “The Mechanosensitive Ion Channel Piezo2 Mediates Sensitivity to Mechanical Pain in Mice.” Science Translational Medicine 10, no. 462:eaat9897. 10.1126/scitranslmed.aat9897. 30305457 PMC6709986

[brb371385-bib-0072] Muthupillai, R. , D. J. Lomas , P. J. Rossman , J. F. Greenleaf , A. Manduca , and R. L. Ehman . 1995. “Magnetic Resonance Elastography by Direct Visualization of Propagating Acoustic Strain Waves.” Nature Medicine 1, no. 6: 601–603. 10.1126/science.7569924 7569924

[brb371385-bib-0073] Nobelförsamlingen . 2021 “The Nobel Assembly at Karolinska Institutet. Discoveries of Receptors For Temperature and Touch.” https://www.nobelprize.org/uploads/2021/10/advanced‐medicine‐2021.pdf.NobelPrize.org. October 2021.

[brb371385-bib-0074] Nordhaug, L. H. , A. Vik , K. Hagen , et al. 2015. “Headaches in Patients With Previous Head Injuries: A Population‐Based Historical Cohort Study (HUNT).” Cephalalgia 36, no. 11: 1009–1019. 10.1177/0333102415618948. 26634833

[brb371385-bib-0075] Obeidat, A. M. , M. J. Wood , N. S. Adamczyk , et al. 2023. “Piezo2 Expressing Nociceptors Mediate Mechanical Sensitization in Experimental Osteoarthritis.” Nature Communications 14, no. 1: 2479. 10.1038/s41467-023-38241-x. PMC10148822 37120427

[brb371385-bib-0076] Overton, C. E. , and R. L. Lipnick . 1991. Studies of Narcosis. Springer.

[brb371385-bib-0077] Paterno, R. , K. A. Folweiler , and A. S. Cohen . 2017. “Pathophysiology and Treatment of Memory Dysfunction after Traumatic Brain Injury.” Current Neurology and Neuroscience Reports 17, no. 7: 52. 10.1007/s11910-017-0762-x. 28500417 PMC5861722

[brb371385-bib-0078] Pathak, M. M. , J. L. Nourse , T. Tran , et al. 2014. “Stretch‐Activated Ion Channel Piezo1 Directs Lineage Choice in human Neural Stem Cells.” Proceedings of the National Academy of Sciences 111, no. 45: 16148–16153. 10.1073/pnas.1409802111. PMC4234578 25349416

[brb371385-bib-0079] Pavuluri, K. , J. M. Scott , J. Huston Iii , et al. 2023. “Differential Effect of Dementia Etiology on Cortical Stiffness as Assessed by MR Elastography.” NeuroImage: Clinical 37: 103328. 10.1016/j.nicl.2023.103328. 36696808 PMC9879983

[brb371385-bib-0080] Pepin, K. M. , K. P. Mcgee , A. Arani , et al. 2018. “MR Elastography Analysis of Glioma Stiffness and IDH1‐Mutation Status.” AJNR American Journal of Neuroradiology 39, no. 1: 31–36. 10.3174/ajnr.A5415. 29074637 PMC5766369

[brb371385-bib-0081] Redell, J. B. , M. E. Maynard , E. L. Underwood , S. M. Vita , P. K. Dash , and N. Kobori . 2020. “Traumatic Brain Injury and Hippocampal Neurogenesis: Functional Implications.” Experimental Neurology 331: 113372. 10.1016/j.expneurol.2020.113372. 32504636 PMC7803458

[brb371385-bib-0082] Rocha, D. N. , E. D. Carvalho , J. B. Relvas , M. J. Oliveira , and A. P. Pêgo . 2022. “Mechanotransduction: Exploring New Therapeutic Avenues in Central Nervous System Pathology.” Frontiers in Neuroscience 16: 861613. 10.3389/fnins.2022.861613. 35573316 PMC9096357

[brb371385-bib-0083] Romero, L. O. , A. E. Massey , A. D. Mata‐Daboin , et al. 2019. “Dietary Fatty Acids Fine‐Tune Piezo1 Mechanical Response.” Nature Communications 10, no. 1: 1200. 10.1038/s41467-019-09055-7. PMC6416271 30867417

[brb371385-bib-0084] Ryu, Y. , A. Wague , X. Liu , B. T. Feeley , A. R. Ferguson , and K. Morioka . 2024. “Cellular Signaling Pathways in the Nervous System Activated by Various Mechanical and Electromagnetic Stimuli.” Frontiers in Molecular Neuroscience 17: 1427070. 10.3389/fnmol.2024.1427070. 39430293 PMC11486767

[brb371385-bib-0085] Sack, I. 2022. “Magnetic Resonance Elastography From Fundamental Soft‐Tissue Mechanics to Diagnostic Imaging.” Nature Reviews Physics 5: 25–42. 10.1038/s42254-022-00543-2.

[brb371385-bib-0086] Sack, I. , B. Beierbach , U. Hamhaber , D. Klatt , and J. Braun . 2013. “Non‐Invasive Measurement of Brain Viscoelasticity Using Magnetic Resonance Elastography.” Physics in Medicine & Biology 58, no. 2: 370–386. 10.1002/nbm.1189 17614101

[brb371385-bib-0087] Segel, M. , B. Neumann , M. F. E. Hill , et al. 2019. “Niche Stiffness Underlies the Ageing of Central Nervous System Progenitor Cells.” Nature 573, no. 7772: 130–134. 10.1038/s41586-019-1484-9. 31413369 PMC7025879

[brb371385-bib-0088] Sinclair, A. J. 2023. “Idiopathic Intracranial Hypertension: A Step Change in Understanding the Disease Mechanisms.” Nature Reviews Neurology 19: 641–642. 10.1038/s41582-023-00893-0 37957260

[brb371385-bib-0089] Singhmar, P. , X. Huo , N. Eijkelkamp , et al. 2016. “Critical Role for Epac1 in Inflammatory Pain Controlled by GRK2‐Mediated Phosphorylation of Epac1.” Proceedings of the National Academy of Sciences 113, no. 11: 3036–3041. 10.1073/pnas.1516036113. PMC4801297 26929333

[brb371385-bib-0090] Solis, A. G. , P. Bielecki , H. R. Steach , et al. 2019. “Mechanosensation of Cyclical Force by PIEZO1 Is Essential for Innate Immunity.” Nature 573, no. 7772: 69–74. 10.1038/s41586-019-1485-8. 31435009 PMC6939392

[brb371385-bib-0091] Song, Y. , D. Li , O. Farrelly , et al. 2019. “The Mechanosensitive Ion Channel Piezo Inhibits Axon Regeneration.” Neuron 102, no. 2: 373–389.e6. 10.1016/j.neuron.2019.01.050. 30819546 PMC6487666

[brb371385-bib-0092] Svensson, S. F. 2023. MR Elastography of the Brain: In Healthy Subjects and Patients With Neurological Disease. University of Oslo.

[brb371385-bib-0093] Terminology . 2025. —International Association for the Study of Pain (IASP). International Association for the Study of Pain. https://www.iasp‐pain.org/resources/terminology/.

[brb371385-bib-0094] Ucar, H. , S. Watanabe , J. Noguchi , et al. 2021. “Mechanical Actions of Dendritic‐Spine Enlargement on Presynaptic Exocytosis.” Nature 600, no. 7890: 686–689. 10.1038/s41586-021-04125-7. 34819666

[brb371385-bib-0095] Ultrasound‐Induced Wireless Implantable Stimulator for Adaptive Pain Management . 2025 Nature Electronics 8, no. 5: 384–385. 10.1038/s41928-025-01377-3.

[brb371385-bib-0096] Velasco‐Estevez, M. , M. Mampay , H. Boutin , et al. 2018. “Infection Augments Expression of Mechanosensing Piezo1 Channels in Amyloid Plaque‐Reactive Astrocytes.” Frontiers in Aging Neuroscience 10: 10:332. 30405400 10.3389/fnagi.2018.00332 PMC6204357

[brb371385-bib-0097] Wan, Y. , H. Wang , X. Fan , et al. 2023. “Mechanosensitive Channel Piezo1 Is an Essential Regulator in Cell Cycle Progression of Optic Nerve Head Astrocytes.” Glia 71: 1233–1246. 10.1002/glia.24334. 36598105

[brb371385-bib-0098] Wan, Y. , J. Zhou , and H. Li . 2024. “The Role of Mechanosensitive Piezo Channels in Chronic Pain.” Journal of Pain Research 17: 4199–4212. 10.2147/JPR.S490459. 39679432 PMC11646438

[brb371385-bib-0099] Weickenmeier, J. 2016. “Brain Stiffness Changes in Hydrocephalus Measured by MR Elastography.” Journal of Neurosurgery 125: 1471–1479.

[brb371385-bib-0100] Yin, Z. , A. J. Romano , A. Manduca , R. L. Ehman , and J. Huston . 2018. “Stiffness and Beyond: What MR Elastography Can Tell Us about Brain Structure and Function Under Physiologic and Pathologic Conditions.” Topics in Magnetic Resonance Imaging 27, no. 5: 305–318. 10.1097/RMR.0000000000000178. 30289827 PMC6176744

[brb371385-bib-0101] Zheng, Q. , H. Liu , W. Yu , et al. 2023. “Mechanical Properties of the Brain: Focus on the Essential Role of Piezo1‐Mediated Mechanotransduction in the CNS.” Brain and Behaviour 13, no. 9:e3136. 10.1002/brb3.3136. PMC10498085 37366640

[brb371385-bib-0102] Zhu, T. , J. Guo , Y. Wu , et al. 2023. “The Mechanosensitive Ion Channel Piezo1 Modulates the Migration and Immune Response of Microglia.” Iscience 26, no. 2:105993. 10.1016/j.isci.2023.105993. 36798430 PMC9926228

